# Intrahepatic macrophage reprogramming associated with lipid metabolism in hepatitis B virus-related acute-on-chronic liver failure

**DOI:** 10.1186/s12967-023-04294-1

**Published:** 2023-06-28

**Authors:** Bo Peng, Hao Li, Kai Liu, Pengpeng Zhang, Quan Zhuang, Junhui Li, Min Yang, Ke Cheng, Yingzi Ming

**Affiliations:** 1 Transplantation Center, The Third Xiangya Hospital, Central South University, Hunan 410013 Changsha, China; 2 Engineering and Technology Research Center for Transplantation Medicine of National Health Commission, Changsha, China

**Keywords:** Acute-on-chronic liver failure, Hepatitis B virus, scRNA-seq, TREM2, Lipid metabolomics, Free fatty acids, Cirrhosis-associated immune dysfunction

## Abstract

**Background:**

Acute-on-chronic liver failure (ACLF) is a severe syndrome with high short-term mortality, but the pathophysiology still remains largely unknown. Immune dysregulation and metabolic disorders contribute to the progression of ACLF, but the crosstalk between immunity and metabolism during ACLF is less understood. This study aims to depict the immune microenvironment in the liver during ACLF, and explore the role of lipid metabolic disorder on immunity.

**Methods:**

Single-cell RNA-sequencing (scRNA-seq) was performed using the liver non-parenchymal cells (NPCs) and peripheral blood mononuclear cells (PBMCs) from healthy controls, cirrhosis patients and ACLF patients. A series of inflammation-related cytokines and chemokines were detected using liver and plasma samples. The lipid metabolomics targeted free fatty acids (FFAs) in the liver was also detected.

**Results:**

The scRNA-seq analysis of liver NPCs showed a significant increase of monocytes/macrophages (Mono/Mac) infiltration in ACLF livers, whereas the resident Kupffer cells (KCs) were exhausted. A characterized TREM2^+^ Mono/Mac subpopulation was identified in ACLF, and showed immunosuppressive function. Combined with the scRNA-seq data from PBMCs, the pseudotime analysis revealed that the TREM2^+^ Mono/Mac were differentiated from the peripheral monocytes and correlated with lipid metabolism-related genes including *APOE, APOC1, FABP5* and *TREM2*. The targeted lipid metabolomics proved the accumulation of unsaturated FFAs associated with α-linolenic acid (α-LA) and α-LA metabolism and beta oxidation of very long chain fatty acids in the ACLF livers, indicating that unsaturated FFAs might promote the differentiation of TREM2^+^ Mono/Mac during ACLF.

**Conclusions:**

The reprogramming of macrophages was found in the liver during ACLF. The immunosuppressive TREM2^+^ macrophages were enriched in the ACLF liver and contributed to the immunosuppressive hepatic microenvironment. The accumulation of unsaturated FFAs in the ACLF liver promoted the reprogramming of the macrophages. It might be a potential target to improve the immune deficiency of ACLF patients through regulating lipid metabolism.

**Supplementary Information:**

The online version contains supplementary material available at 10.1186/s12967-023-04294-1.

## Background

Acute-on-chronic liver failure (ACLF) is a syndrome associated with high short-term mortality in patients with acutely decompensated cirrhosis [1]. Although improvements have been achieved in the management of viral hepatitis, it remains the leading cause of cirrhosis worldwide, and the rising prevalence of non-alcoholic fatty liver disease (NAFLD) and alcohol-associated cirrhosis further increases the burden of cirrhosis [2]. Among patients admitted with decompensated cirrhosis, the prevalence of ACLF is as high as 35% [3]. The overall 28-day mortality of ACLF worldwide is 45% [3], and for hepatitis B virus (HBV)-ACLF specifically, the 28-day mortality is 60.2% for non-cirrhotic HBV-ACLF and 52.1% for cirrhotic HBV-ACLF, respectively [4]. The grading of ACLF, which is determined by the number of organ failure, is closely associated with the mortality and prognosis [5]. The main principle of treatment is to diagnose and treat precipitating event and provide supportive therapy [1]. Prompt anti-infective therapy, immediate nucleos(t)ide analogue treatment for HBV-related ACLF and prednisolone for severe alcoholic hepatitis, use of artificial liver support systems are important treatment options, and liver transplantation is considered as the irreplaceable and definitive treatment for ACLF, particularly for patients who do not improve with supportive care [6, 7].

As a complicated and heterogenous syndrome, the pathophysiology of ACLF still remains largely unknown. In fact, controversies exist for ACLF, and the definitions and diagnostic criteria differ in different studies (the widely-recognized definitions and diagnostic criteria of ACLF were thoroughly reviewed in Ref 1) [1]. At present, the recognized features of ACLF that make it a separate clinical entity are intense systemic inflammation, pro-inflammatory precipitating events and single- or multiple-organ failures [1]. The most frequent precipitating events are infections and acute alcoholic hepatitis for the patients in Western countries and Hepatitis B relapse for the HBV-related patients in China [1, 4]. Generally, the precipitating events are the initial factors to induce the pathogen-associated molecular patterns (PAMPs) and/or damage-associated molecular patterns (DAMPs), thus leading to systemic inflammation and subsequent organ failure. Notably, immune deficiency occurs simultaneously with systemic inflammation, leading to inability to eliminate pathogens, especially derived from the gut [8]. In fact, cirrhosis-associated immune dysfunction (CAID) begins in compensated cirrhosis, progresses through the decompensated stage and peaks in ACLF [9, 10]. The impaired immune system makes ACLF patients more susceptible to bacterial infection, which in turn is one of the most critical triggers for ACLF [8]. Therefore, it is important and urgent to further explore the mechanism for the immune dysregulation in ACLF.

The crosstalk between immunity and metabolism, namely immune-metabolism, has been highlighted in recent studies [11]. Due to the highly intense systemic inflammation in ACLF, a rapid and widespread catabolism is induced to fuel the energetically expensive immune responses. As a result, profound metabolic alterations in glycometabolism, lipid metabolism, and amino acid metabolism have been found in ACLF, and the metabolic disorders further contribute to organ dysfunction/failure [12]. Both the metabolomics and transcriptomics analyses reveal the immune-metabolism disorders in the peripheral blood of ACLF, and the metabolite fingerprint and the key differentially expressed genes have been identified [13–17]. Through regulating the key metabolic pathway and genes, it may interrupt the vicious circle during the progression of ACLF and improve the prognosis.

The cutting-edge single-cell RNA-sequencing (scRNA-seq) technology can provide high-definition and high-throughput view on single-cell level. Although some limitations exist, which includes lack of information at protein level and high requirement on statistical processing, it is still a powerful tool for us to reveal the intricate mechanisms under ACLF. In our previous work, we reported the dysfunction of lymphatic endothelial cells caused by the secreted phosphoprotein 1 from infiltrated monocytes/macrophages in ACLF using scRNA-seq technology [18]. Here, we depicted the unique immune microenvironment in the liver of ACLF using scRNA-seq, and found the characterized monocytes/macrophages subpopulations in ACLF. Through combination of the scRNA-seq dataset from peripheral blood, the differentiation of infiltrated monocytes was revealed. The lipid metabolic disorder in the liver and its role on monocyte differentiation was also studied.

## Materials and methods

### Study population and collection of human samples

The present study included 10 human liver samples which referred to 2 healthy controls (HCs), 3 cirrhosis patients and 5 ACLF patients. The detailed clinical information of the recruited patients was shown in Additional file 1: Table S1. Liver samples from HCs were the residual biopsy specimens of the liver grafts from donation after citizen’s death (China category I, organ donation after brain death) for liver transplantation. Liver samples from cirrhosis and ACLF patients were the resected livers during liver transplantation. The inclusion criteria of the cirrhosis and ACLF patients were as followings: [1] All the patients were HBV-related. [2] Patients received liver transplantation in our center. [3] The ACLF patients met the diagnostic criteria of both the European Association for the Study of the Liver-Chronic Liver Failure Consortium (EASL-CLIF) ACLF definition and the Chinese Group on the Study of Severe Hepatitis B-ACLF (COSSH-ACLF) definition (Additional file 1: Figure S1). The exclusion criteria were as followings: [1] Patients were complicated with HCV infection or other hepatitis viruses. [2] Patients were diagnosed with hepatocellular carcinoma. The study protocol was approved by the Ethics Committee of the Third Xiangya Hospital, Central South University (No. 2020-S023). Written informed consent was obtained from all patients.

### Liver sample preparation and scRNA-seq analysis

The liver non-parenchymal cells (NPCs) were isolated from the fresh liver samples as previously reported [19]. Briefly, liver samples were minced into 1–2 mm pieces and digested with 2 ml GEXSCOPE^®^ Tissue Dissociation Solution (Singleron Biotechnologies) at 37 ℃ for 15 min. After filtration with 70 μm sterile strainers, samples were centrifuged at 50 g for 5 min to remove hepatocytes [19]. The supernatant was centrifuged at 300 g for 5 min, and the precipitation was almost liver NPCs with less hepatocytes (Additional file 1: Figure S2). 100 μl of single-cell NPC suspension or peripheral blood mononuclear cells (PBMCs) at the concentration of 1 × 10^5^ cells/mL was loaded onto the microfluidic devices. scRNA-seq libraries were constructed using the Singleron GEXSCOPE^®^ protocol by GEXSCOPE^®^ Single-Cell RNA Library Kit (Singleron Biotechnologies) as previously described [20]. In short, single-cell suspension was pipetted on the array and incubated for 15 min. Following cell loading, mRNA capture beads (Macosko-2011-10, ChemGenes) were pipetted onto the array and loaded by gravity into microwells. The freeze–thaw lysis buffer was then pipetted on the array and the array was incubated for 5 min at room temperature. A glass slide was used carefully to seal the array and secured using a manual clamp (Agilent, G2534A). Three freeze–thaw cycles were performed to lyse the cells, freezing cells in −80 °C freezer or dry ice/ethanol bath for 10–15 min and thawing them at room temperature for 10–15 min. Following lysis, microwell array was incubated for an hour inside a wet chamber for mRNA capture onto beads. mRNA binding occurred in the freeze–thaw lysis buffer without the need for buffer exchange. After incubation, the array was interfaced with a lifter slip and transferred to a 4-well plate filled with phosphate-buffered saline (PBS) in inverted orientation. The 4-well plated was centrifuged at 1000 g for 5 min to release the beads into the PBS solution. Any remaining beads were carefully removed by scraping the array surface gently with a glass slide or a pipette tip. The PBS solution containing the beads were transferred to a 15 ml falcon tube, centrifuged at 1000 g for 5 min and transferred to a 1.5 ml Eppendorf tube in 1 ml volume to proceed with reverse transcription to build libraries.

Individual libraries were diluted to 4 nM and pooled for sequencing. Pools were sequenced on Illumina HiSeq X with 150 bp paired end reads. Raw reads were processed with fastQC and fastp to remove low quality reads. Poly-A tails and adaptor sequences were removed by cutadapt. After quality control, reads were mapped to the reference genome GRCh38 (ensembl version 92 annotation). Gene counts and unique molecular identifier (UMI) counts were acquired by featureCounts software. Expression matrix files for subsequent analysis were generated based on gene counts and UMI counts. Cells were filtered by gene counts greater than 200 and UMI counts below 6,000. Cells with over 25% mitochondrial content were removed.

After filtering, 26,400 cells and 24,194 genes were retained for the downstream analyses. Seurat v4.0 was used for dimension-reduction and clustering [21]. To investigate the potential functions of each cluster, Kyoto encyclopedia of genes and genomes (KEGG) and gene ontology (GO) analyses were used with the “enrichplot” R package version. The highly expressed genes in each cell subpopulation (defined as differences greater than 2^0.5^ and P < 10^–50^ or P < 10^–10^) were selected and compared with the genes in each pathway in the KEGG and GO databases to select the most reliable pathway and annotate the cell function. The signature score of pro-inflammatory/anti-inflammatory genes was assessed using Seurat v4.0 R package. To map differentiation/conversion of monocytes/macrophages cell subtype, pseudotime trajectory analysis was performed with Monocle2 and Monolce3**.** In short, we ordered cells in a semi-supervised manner on the basis of their Seurat clustering, and used the top 500 variable genes as input and mapped them onto UMAP or pseudotime trajectory visualizations.

### Immunofluorescence staining

The Alexa Fluor^™^ 488 Tyramide SuperBoost^™^ Kit (B40922, Invitrogen) was used for immunofluorescence staining as previously described [22]. Briefly, liver samples were fixed in 10% neutralized buffered formaldehyde at 4 ºC for 48 h. After retrieving antigens, the sections were incubated with the primary antibody overnight at 4 ℃. The secondary antibody was incubated for 1 h and the third antibody was used to develop fluorescence. Then the sections were incubated with 10 mM EDTA buffer (pH 6.0) for 15 min at approximately 100 ºC to inactivate horseradish peroxidase, and the primary antibody targeted another antigen was incubated overnight at 4 ℃. The fluorescent second antibody was incubated for 1 h to develop fluorescence. Images were obtained using Leica Microsystems DMI 3000B observer. For positive signal quantification, at least 5 images X100 and X200 magnification were obtained per slide.

### Cytokines and chemokines assessment

The Quantibody^®^ array kit (Raybiotech) was used to quantify a series of cytokines and chemokines in the plasma and liver samples from ACLF and cirrhosis patients as previously reported [23]. Interleukin (IL)-1α, IL-1β, IL-4, IL-6, IL-8, IL-10, IL-13, monocyte chemoattractant protein-1 (MCP-1), interferon (IFN)-γ and tumor necrosis factor (TNF)-α were included in the kit. Before liver transplantation, the peripheral blood of ACLF and cirrhosis patients was collected in sterile tubes with EDTA. Fresh blood was centrifuged immediately at 1500 round per minute (rpm) for 15 min at 4 ℃, and upper plasma was collected and stored at −80 ℃ before the test. The diseased livers of the patients were divided into small tissue blocks, rinsed in ice-cold saline to remove blood and connective tissue. After drying with filter paper, the tissue blocks were weighed and quickly cut into pieces. Samples were then fully ground in cold PBS on ice to prepare 10% tissue homogenate and centrifuged at 3000 rpm for 15 min at 4 ℃. The clear liquid on the upper layer was collected for test. The Quantibody^®^ array test was performed according to the manufacture's instruction.

### Lipid metabolomics detection and analysis

The targeted lipid metabolites including 41 free fatty acids (FFAs) of the liver samples were quantified using an ultra-performance liquid chromatography-tandem mass spectrometry (UPLC-MS/MS) system (ACQUITY UPLC-Xevo TQ-S, Waters Corp., Milford, MA, USA). Briefly, 10 liver samples from HBV-related cirrhosis patients and 10 liver samples from ACLF patients were harvested and stored in an Eppendorf Safelock microcentrifuge tube. Each sample was mixed with 10 pre-chilled zirconium oxide beads and homogenated for 3 min. 120 μL of methanol containing internal standard was added to extract the metabolites and then centrifuged at 18,000 g for 20 min. Then the supernatant was used for LC–MS analysis.

Raw data files generated by UPLC-MS/MS were processed using MassLynx software (v4.1, Waters, Milford, MA, USA) for peak integration, correction and quantification. Standard score (Z-score) was used to standardize the amount of metabolites. The metabolites were compared using Mann–Whitney U test and showed by volcano plot and bubble chart. Finally, the pathway-associated metabolite sets were used for pathway enrichment analysis.

### Monocyte selection, culture and stimulation assay

THP-1 cells were purchased from Procell Life Science&Technology and cultured in THP-1 cell-specific medium (Procell, CM-0233) containing 10% fetal bovine serum (FBS, Procell, 164210-50), 0.05 mM β-mercaptoethanol (Procell, PB180633) and 1% Penicillin–Streptomycin (Procell, PB180120) at 37 °C in a 5% CO_2_ environment. 320 nmol/L phorbol 12-myristate 13-acetate (PMA, Sigma-Aldrich, 16561-29-8) was added to incubate THP-1 cells for 18 h, and then changed to PMA-free medium to incubate cells for 24 h to obtain resting macrophages. Thereafter, 100 nmol/L PMA, 100 ng/ml lipopolysaccharide (LPS, Sigma-Aldrich, L2880) and 20 ng/ml IFN-γ (Peprotech, 300-02) or 100 nmol/L PMA, 20 ng/ml IL-4 (abbkine, PRP2017) and 20 ng/ml IL-13 (abbkine, PRP100108) were added to THP-1 medium, respectively. After incubation for 48 h, the resting macrophages were induced to differentiate into M1 type or M2 type macrophages.

Human peripheral CD14^+^ monocytes were positively selected from PBMCs of eight HCs using magnetic activated cell sorting (MACS) CD14 microbeads (Miltenyi Biotec, 130-050-201). The selected CD14^+^ monocytes were stimulated with unsaturated FFA as previously reported [24]. Briefly, the CD14^+^ monocytes were divided into four groups: [1] unstimulated group: cells were directly detected; [2] LPS group: cells were stimulated with 100 ng/mL LPS for 24 h; [3] LPS + α-linolenic acid (α-LA, Sigma-Aldrich, L-039) group: cells were pretreated with 60 μM α-LA for 2 h, and then 100 ng/mL LPS was added and cells were cultured for 24 h; [4] α-LA group: cells were stimulated with 60 μM α-LA for 24 h. After stimulation, cells were collected for flow cytometry (FCM) analysis.

### Real-time quantitative PCR

Total RNA from polarized macrophages was obtained using the SteadyPure Rapid RNA Extraction Kit (Accurate Biology, AG21023), and the total RNA was quality-checked and quantified by NanoDrop 2000 (ThermoFisher Scientific). Evo M-MLV Reverse Transcription Master Mix Kit (Accurate Biology, AG11728) was used to remove genomic DNA and reverse transcription. PCR were performed using 2X Universal SYBR Green Fast qPCR Mix (ABclonal, RK21203) on the LightCycler^®^ 480 System (Roche). All experimental steps refer to manufacturer's instructions, and the primers used were synthesized by Sangon Biotech.

### FCM analysis

The selected monocytes were collected and washed by Dulbecco’s phosphate-buffered saline (DPBS) twice. Then cells were stained with Zombie Violet^™^ fixable viability dye (BioLegend, 423113) for 15 min. Without washing the cells, the cocktails of anti-CD14-PerCP-Cyanine5.5 (Clone 61D3, Invitrogen) and anti-TREM2-APC (Clone 237920, R&D) were added and cells were incubated in dark at room temperature for another 15 min. After washing, cells were detected using BD FACSCanto II. The results were analyzed using Flowjo 10.4 (Becton, Dickinson & Company, USA).

### ***Statistical analysis***

All data were expressed as mean ± standard deviation (SD) or standard error of mean (SEM). The comparison between two groups was performed using unpaired Student’s t-test, Welch’s t test or the Mann–Whitney U test, where appropriate. The comparison of median fluorescence intensity (MFI) of TREM2 on monocytes was performed with paired t-test. Categorical data were compared using Pearson’s chi-squared (χ^2^) test or Fisher’s exact test, where appropriate. The comparison between multiple groups was performed using one-way ANOVA. Statistical analysis was performed using SPSS version 22.0 (SPSS, Inc., Chicago, IL, USA). A P value of < 0.05 was considered to be statistically significant.

## Results

### scRNA-seq revealed a significant increase of monocyte/macrophage infiltration into the livers of ACLF patients

NPCs isolated from liver samples of 2 HCs, 3 cirrhosis patients and 5 ACLF patients were analyzed by scRNA-seq, and the dataset was reported in our previous work [18]. The clinical information of the patients was shown in Additional file 1: Table S1. After removing the suspicious double cells and low-activity cells through quality control, a total of 26,200 cells were included in the further analysis. These cells were re-clustered into 20 subpopulations which were annotated by the high expression genes and the classic immune cell markers in each cluster (Additional file 1: Figure S3a–c). Finally, these 20 subpopulations were annotated as T cells, NK cells, monocytes/macrophages (Mono/Mac), endothelial/epithelial cells (Endo/Epi), dendritic cells (DC), B cells, neutrophils (Neu), erythrocytes, mast cells, fibroblasts and platelets (Additional file 1: Figure S3d). When shown by group, the scRNA-seq data revealed a significant increase of Mono/Mac (clusters 2, 7, 8 and 17 which were CD14 and CD68 co-expressed) in the ACLF group compared with the cirrhosis group (Additional file 1: Figure S3e). CD68 immunofluorescence staining of the liver samples further confirmed a significant increase of Mono/Mac infiltration in the ACLF liver compared with the HC and cirrhosis liver (Additional file 1: Figure S3f).

### ***ACLF patients showed distinct features of intrahepatic Mono/Mac***

**Fig. 1:**
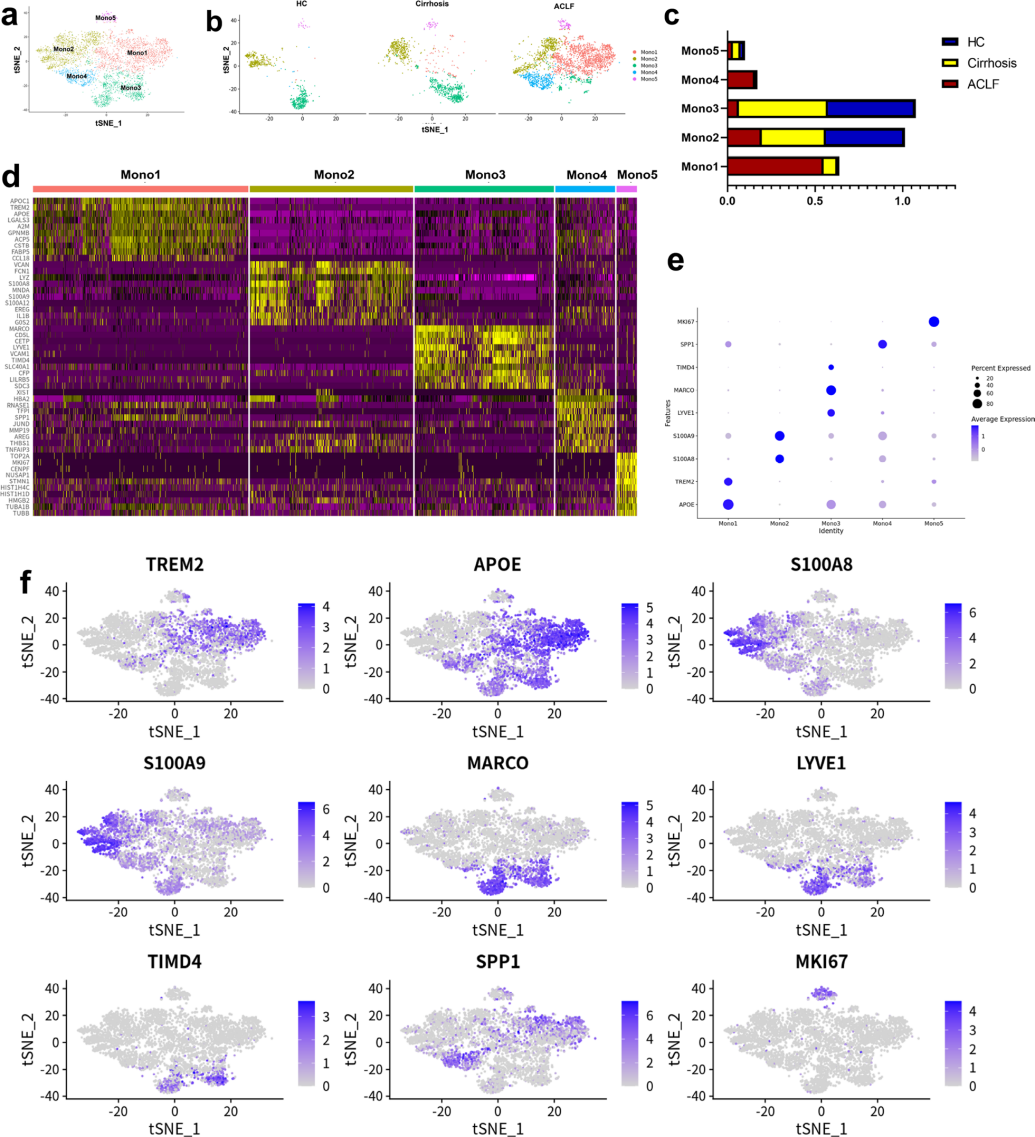
The scRNA-seq analysis of monocytes/macrophages in the livers. **a** 4738 monocytes/macrophages in the livers were re-clustered into 5 clusters according to their gene expression using Seurat v4.0 and the result was shown by tSNE.** b** The distribution of 5 monocytes/macrophages clusters among HCs, cirrhosis and ACLF patients. Clusters Mono1 and Mono4 were characteristically enriched in ACLF livers.** c** The proportions of 5 monocytes/macrophages clusters among HCs, cirrhosis and ACLF patients. The HCs and cirrhosis patients had similar proportions of each cluster, while the ACLF patients had high proportions of Mono1. **d** The heat map of the DE genes for each cluster. **e** The dotplot of the representative marker genes for each cluster. **f** The tSNE plots of marker genes for each cluster

Due to a significant increase of Mono/Mac infiltration in ACLF livers, the characteristic of Mono/Mac subpopulations was further analyzed. Totally, 4738 Mono/Mac were re-clustered into 5 clusters (Mono1—Mono5, Fig. 1a) according to their gene expression (Fig. 1d and e). The HCs and cirrhosis patients showed a similar intrahepatic Mono/Mac subpopulation feature, but in contrast, the feature of the intrahepatic Mono/Mac in the ACLF patients was distinct. Generally, clusters Mono1 and Mono4 were characteristically enriched in the ACLF group, while the ratios of Mono2 and Mono3 in the ACLF group were decreased when compared with the HCs and cirrhosis patients (Fig. 1b and c).

Cluster Mono1 was characterized by enriched expression of *APOC1, TREM2, APOE* (Top DE genes: *APOC1, TREM2, APOE, LGALS3, A2M, GPNMB, ACP5, CSTB, FABP5, CCL18*, Fig. 1d and f), suggesting that this cluster played a role in lipid metabolism and immune regulation, which was in accordance with the KEGG and GO analyses (Additional file 1: Figure S4a). Another ACLF characterized cluster was Mono4, which was enriched in the expression of *TFPI, SPP1, JUND* (Top DE genes: *XIST, HBA2, RNASE1, TFPI, SPP1, JUND, MMP19, AREG, THBS1, TNFAIP3*, Fig. 1d and f). The DE genes in this cluster and the KEGG and GO analyses suggested that this cluster was related to oxidative stress and apoptosis (Additional file 1: Figure S4b). Cluster Mono2 was characterized by enriched expression of *LYZ, S100A8, S100A9*, which was similar to the classic monocytes. Cluster Mono3, characterized by *MARCO, LYVE1, TIMD4*, was the subpopulation of classic Kupffer cells (KCs). Cluster Mono5, which had similar ratios in three groups, was characterized by *MKI67* and represented the proliferative cells.

**Fig. 2:**
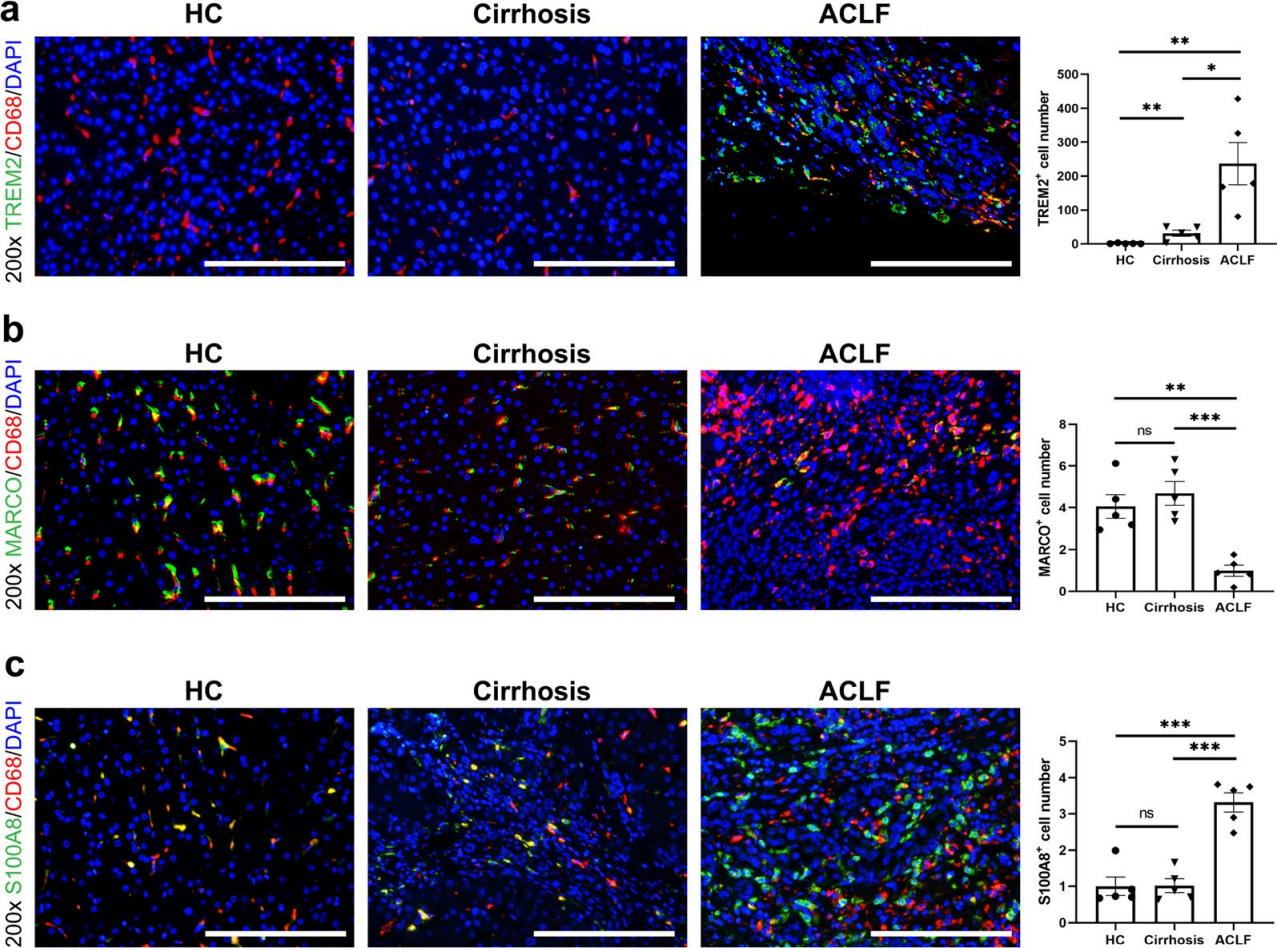
The representative immunofluorescence images of monocytes/macrophages clusters in the livers of HCs, cirrhosis and ACLF patients. **a** The immunofluorescence images (n = 5) of CD68 (red), TREM2 (green) and DAPI (blue) in the livers. The ACLF patients had much higher number of TREM2^+^ cells in the livers compared with HCs and cirrhosis patients. **b** The immunofluorescence images (n = 5) of CD68 (red), MARCO (green) and DAPI (blue) in the livers. The HCs and cirrhosis patients had similar number of MARCO^+^ cells, while the ACLF patients showed significant decrease of them. **c** The immunofluorescence images (n = 5) of CD68 (red), S100A8 (green) and DAPI (blue) in the livers. The ACLF patients had higher number of S100A8.^+^ cells in the livers compared with HCs and cirrhosis patients. For all images, the positive signal number of each sample was determined by the mean value of the TREM2/MARCO/S100A8 positive signals in five random fields after data standardization. (ns not significant, *p < 0.05, **p < 0.01, ***p < 0.001, bar = 200 μm)

Immunofluorescent staining was performed to further confirm the subpopulations of Mono/Mac in the liver samples from HC, cirrhosis and ACLF groups. There was a significantly increased infiltration of TREM2^+^ Mono/Mac in the livers of ACLF group, but few of them existed in the livers of HC group (Fig. 2a). In contrast, the MARCO^+^ KCs were exhausted in the ACLF group, and the cell counts of KCs in the HC and cirrhosis groups were higher (Fig. 2b). Although the ratio of S100A8^+^ Mono/Mac (cluster Mono2) was decreased in the ACLF group, the absolute cell count of S100A8^+^ Mono/Mac was higher in the ACLF group compared with the HC and cirrhosis groups (Fig. 2c). In accordance with the ratios of Mono2 and Mono3 in the HC and cirrhosis groups, the absolute cell counts of MARCO^+^ KCs and S100A8^+^ Mono/Mac showed no significant difference (Figs. 1c, 2b and c).

### ***TREM2***^+^***Mono1 contributed to immunosuppressive environment in ACLF livers***

**Fig. 3:**
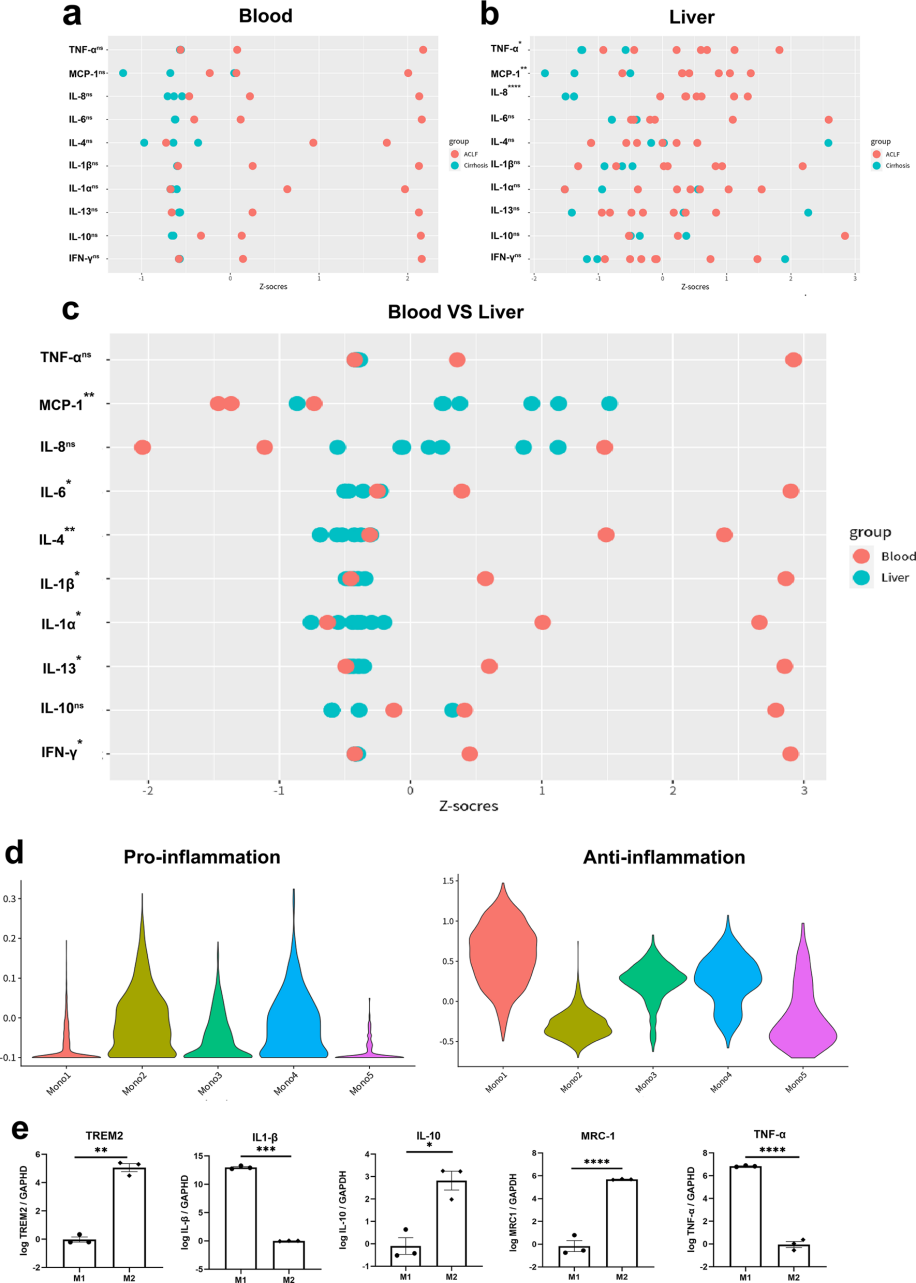
The function of TREM2.^+^ monocytes/macrophages. **a** The cytokines and chemokines detected in the peripheral blood of cirrhosis and ACLF patients using the Quantibody^®^ array kit. The raw data was transformed into standard score and compared with Student’s t-test.** b** The cytokines and chemokines detected in the livers of cirrhosis and ACLF patients using the Quantibody^®^ array kit. The raw data was transformed into standard score and compared with Student’s t-test. **c** The comparison of the cytokines and chemokines between the peripheral blood and livers of ACLF patients. **d** The functional assessment of monocytes/macrophages clusters with pro-inflammatory/anti-inflammatory macrophage genes using Seurat v4.0 R package. The associated genes were list in Additional file 1: Table S2. **e** RNA expression of TREM2, IL-1β, IL-10, MRC-1 and TNF-α in M1 and M2 induced from THP-1 cells in vitro. The expression of TREM2 was much higher in M2. (ns not significant, *p < 0.05, **p < 0.01, ***p < 0.001, ****p < 0.0001)

ACLF was characterized by intense systemic inflammation. Meanwhile, immunosuppression was induced simultaneously in response to inflammation, which increased the susceptibility to infection. We detected a series of inflammation-related cytokines and chemokines in the samples of livers and plasma from ACLF and cirrhosis patients (Fig. 3a and b). A higher trend of pro-inflammatory cytokines including IL-1α, IL-1β, IL-6, IL-8, IFN-γ and TNF-α was detected in the plasma from ACLF patients than that from cirrhosis patients, as well as the anti-inflammatory cytokines including IL-4, IL-10 and IL-13. However, in the liver samples, only TNF-α, MCP-1 and IL-8 were significantly increased in the ACLF group. When compared the results of blood and liver samples from the ACLF group, the liver samples also showed less severe inflammation except for TNF-α, MCP-1 and IL-8 (Fig. 3c).

Because the main infiltrating immune cells in the ACLF livers were Mono/Mac and they showed reprogramming between ACLF and cirrhosis groups, we assessed the function of Mono/Mac clusters to find the potential factor that contributed to the relatively immunosuppressive environment in the ACLF livers. Based on the pro-inflammatory/anti-inflammatory macrophage genes (Additional file 1: Table S2) [25, 26], we evaluated the signature score of each Mono/Mac cluster (Fig. 3d). Cluster Mono2 showed a typical pro-inflammation feature, suggesting it as an inflammatory monocyte subpopulation. Compared with the HC and cirrhosis groups, the ratio of cluster Mono2 was decreased in the ACLF group. In contrast, the ACLF characterized Mono/Mac cluster Mono1 had a typical anti-inflammation feature, indicating it as an immunosuppressive phenotype.

Because cluster Mono1 was predominant in the ACLF livers, we further assessed the function of TREM2^+^ Mono1 in vitro. THP-1 cells were firstly stimulated with PMA, and then induced under M1 (LPS, IFN-γ) or M2 (IL-4, IL-13) condition. TREM2 expression was significantly increased in M2, as well as the immunosuppressive cytokine IL-10 and the M2 typical marker mannose receptor C-1 (MRC-1, CD206). On the contrary, the expression of pro-inflammatory cytokines TNF-α and IL-1β was much lower in TREM2^+^ M2 cells (Fig. 3e).

In summary, the TREM2^+^ Mono1 cluster showed immunosuppressive function, and the increased ratio of Mono1/Mono2 contributed to the immunosuppressive environment in ACLF livers.

### The increase of intrahepatic Mono/Mac in ACLF was due to the infiltration of peripheral blood monocytes

**Fig. 4:**
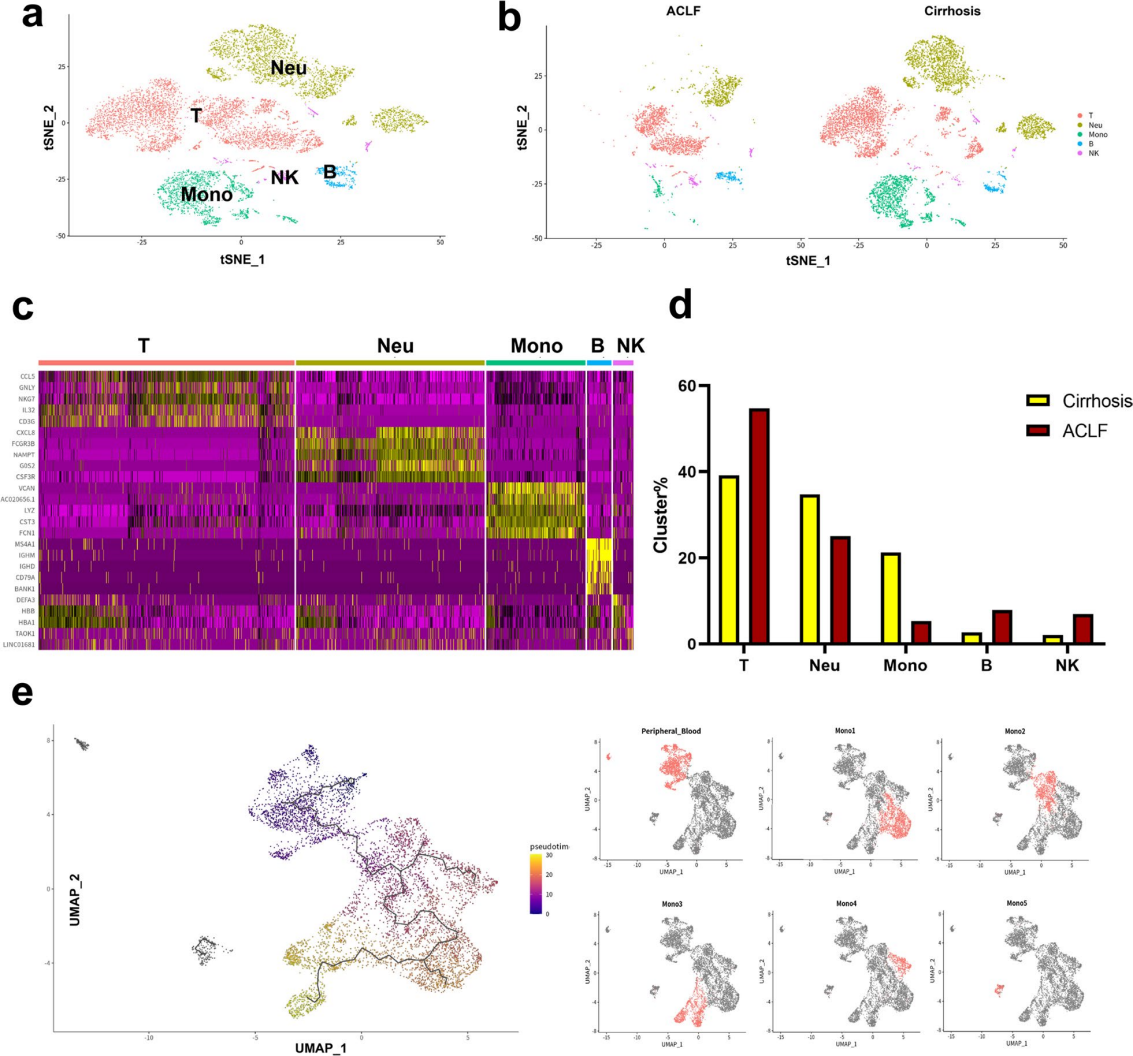
The scRNA-seq analysis of peripheral blood of cirrhosis and ACLF patients. **a** 10,268 PBMCs from cirrhosis and ACLF patients were divided into 5 clusters according to their gene expression using Seurat v4.0. Clusters were annotated as T cells (T), NK cells (NK), B cells (B), monocytes (Mono) and neutrophils (Neu) based on the classic marker genes. **b** The tSNE plot of the 5 clusters in cirrhosis and ACLF patients, respectively. **c** The heat map of representative marker genes of the 5 clusters in PBMCs. **d** The proportions of the 5 clusters in cirrhosis and ACLF patients. The proportion of monocytes was decreased in the peripheral blood of ACLF patients. **e** The pseudotime trajectory (black line) of the intrahepatic monocytes/macrophages and peripheral monocytes shown by UMAP

The intrahepatic resident macrophages, namely KCs (Mono3), showed exhaustion in ACLF livers, but the overall intrahepatic Mono/Mac were significantly increased. To investigate whether the intrahepatic Mono/Mac originated from the peripheral blood monocytes or the resident macrophages, scRNA-seq of PBMCs from 2 ACLF patients and 1 cirrhosis patient was also performed. 10268 PBMCs were annotated into T cells, B cells, NK cells, monocytes and neutrophils based on the high expression genes (Fig. 4a and c). Interestingly, the ratio of monocytes to PBMCs in ACLF patients was decreased compared with that in cirrhosis patients (Fig. 4b and d). Pseudotime analysis based on the combined dataset of peripheral blood monocytes and intrahepatic Mono/Mac was performed to assess the origin of intrahepatic Mono/Mac, and the pseudotemporal trajectory was mapped (Fig. 4e). The differentiation trajectory showed that cluster Mono2 was derived from the peripheral blood monocytes, indicating it as tissue monocytes, which was in accordance with its functional analysis. Cluster Mono2 further differentiated into either cluster Mono1 or Mono4, with no differentiation from cluster Mono3 (KCs) to cluster Mono1 or Mono4. These results suggested that the increase of intrahepatic Mono/Mac was due to the infiltration of peripheral blood monocytes, but not the proliferation of resident macrophages.

### Increase of unsaturated FFAs promoted the macrophage reprogramming in ACLF livers

**Fig. 5:**
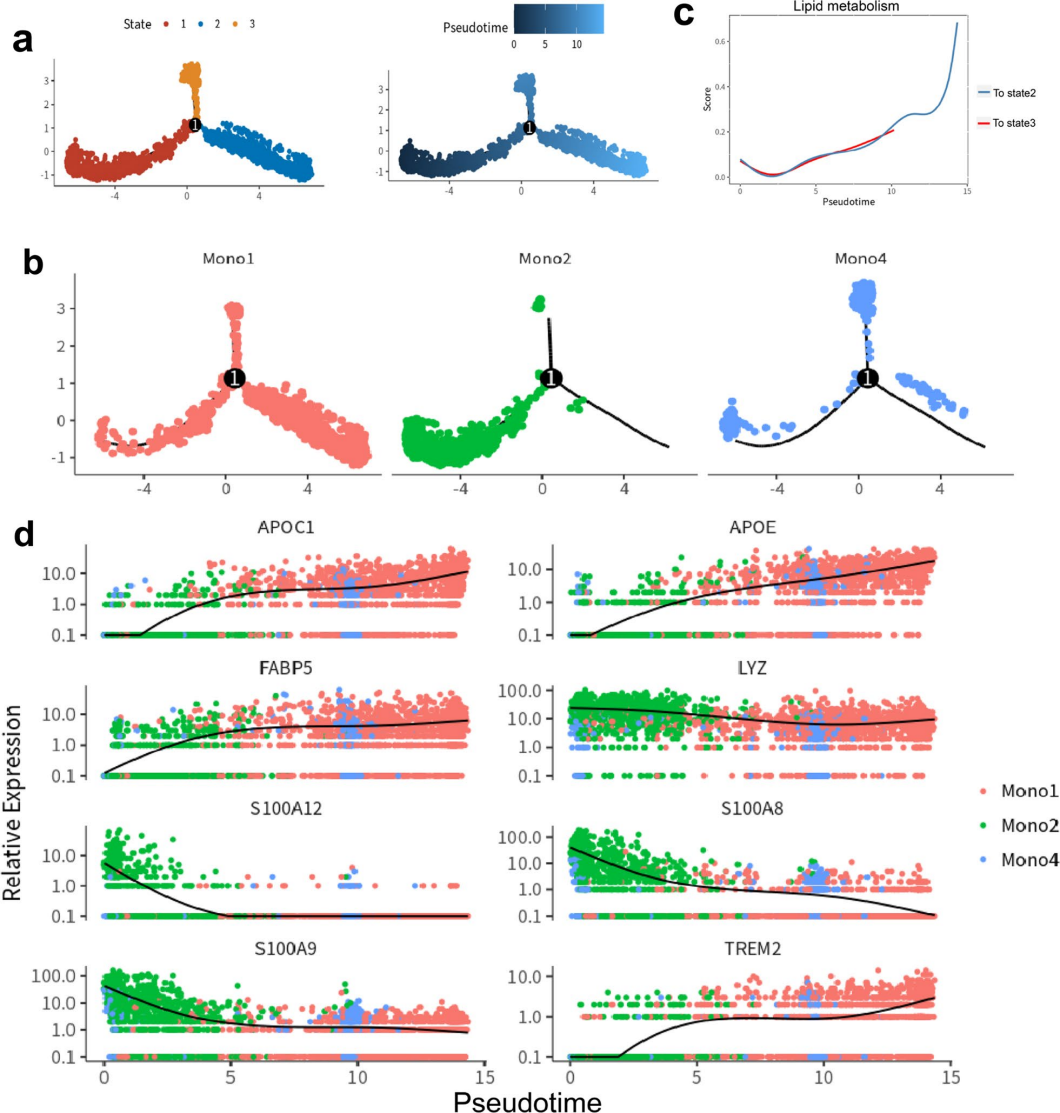
The pseudotime analysis of monocytes/macrophages. **a** The pseudotime trajectory of Mono1, Mono2 and Mono4. All cells were clustered into 3 states. The No.1 point represent as the differentiation point in pseudotime differentiation. **b** The distribution of monocytes/macrophages clusters in the pseudotime trajectory. Mono2 was at the beginning of the trajectory path, and the majority cells of clusters Mono1 and Mono4 were at the terminals of the bifurcation, respectively **c** Changes in lipid metabolism scores along the direction of pseudotime trajectory. **d** The expression of key genes along the pseudotime trajectory. Genes related to lipid metabolism, including *APOC1, APOE, FABP5* and *TREM2* were increased, while genes related to inflammation, including *LYZ, S100A12, S100A8* and *S100A9* were decreased

To further characterize the differentiation of tissue monocytes, the pseudotime analysis on clusters Mono1, Mono2 and Mono4 was performed. The result showed a three-branch trajectory with 3 distinct cell states (Fig. 5a). Cluster Mono2 was at the beginning of the trajectory path, and the majority cells of clusters Mono1 and Mono4 were at the terminals of the bifurcation, respectively (Fig. 5b). Along the differentiation, the expression of genes related to lipid metabolism was significantly increased in cluster Mono1 (Fig. 5c). The gene heat map with spline curves detailed the differentially expressed genes along monocyte differentiation (Fig. 5d). It showed that genes related to lipid metabolism, including *APOE, APOC1, FABP5* and *TREM2*, were upregulated along the pseudotemporal trajectory from Mono2 to Mono1, which was in accordance with gene enrichment analysis (Additional file 1: Figure S5).

**Fig. 6:**
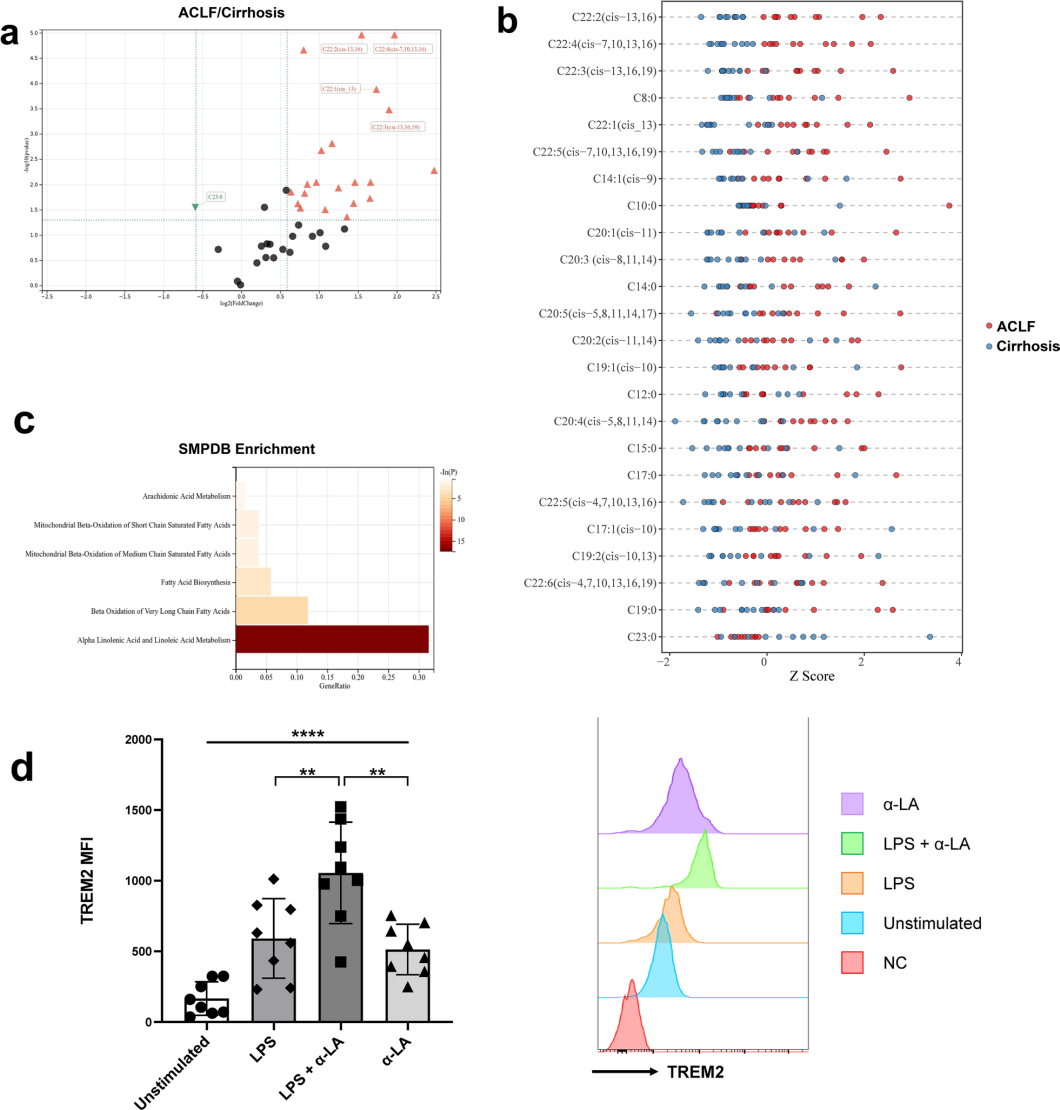
The targeted lipid metabolomics in the livers of cirrhosis and ACLF patients and stimulation test with α-LA. **a** The volcano plot of FFAs in the livers showed as the ACLF group over the cirrhosis group. 23 FFAs were increased in ACLF group while only 1 FFA was increased in cirrhosis group. **b** The 24 differentiated FFAs for each sample shown by Z score. **c** The metabolite sets enrichment analysis for the increased FFAs in ACLF livers. **d** The expression of TREM2 on selected CD14^+^ peripheral monocytes stimulated in vitro. The expression of TREM2 was significantly increased when stimulated with α-LA (60 μM) and LPS (100 ng/mL). (**p < 0.01, ****p < 0.0001, tested by paired t-test)

The upregulation of lipid metabolism-related genes during Mono2 differentiation suggested that certain lipids might promote monocyte reprogramming in ACLF. To investigate whether lipid metabolic disorder existed in ACLF livers, lipid metabolomics targeted FFAs was detected using liver samples from 10 ACLF patients and 10 cirrhosis patients. A total of 41 FFAs were detected. Twenty-three FFAs, most of which were unsaturated FFAs, were higher in the ACLF livers whereas only 1 FFA [tricosanoic acid (C23:0)] was higher in the cirrhosis livers (Fig. 6a and b). Among them, docosadienoic acid [C22:2 (cis-13, 16)], docosatetraenoic acid [C22:4 (cis-7, 10, 13, 16)] and docosatrienoic acid [C22:3 (cis-13, 16, 19)] showed the most significant difference. The metabolite sets enrichment analysis for the increased FFAs in ACLF livers indicated that they were associated with α-LA and α-LA metabolism and beta oxidation of very long chain fatty acids (Fig. 6c). Therefore, the increase of unsaturated FFAs and the disorder of α-LA metabolism might promote the differentiation of TREM2^+^ Mono/Mac in ACLF livers.

To validate the potential promotive effects of unsaturated FFAs on TREM2 expression, the in vitro stimulation test of monocytes was performed. Human peripheral CD14^+^ monocytes were positively selected and stimulated with α-LA, LPS or α-LA + LPS. As shown in Fig. 6d, peripheral CD14^+^ monocytes had low expression of TREM2 without stimulation (unstimulated group: 166.1 ± 118.2). When the monocytes were stimulated with LPS or α-LA, the expression of TREM2 slightly increased, but these two groups showed no significant difference (LPS group vs. α-LA group, 591.1 ± 281.1 vs. 513.3 ± 178.8, P = 0.230). Only when the monocytes were stimulated with α-LA under the inflammatory environment, just similar to that in ACLF, the expression of TREM2 significantly increased (LPS + α-LA group: 1055.8 ± 358.4, P = 0.008 for the comparison with LPS group and P = 0.003 for the comparison with α-LA group). Therefore, these results directly proved that the accumulation of α-LA could promote the expression of TREM2 on Mono/Mac under inflammatory environment.

In addition, the expression of CD14 was also detected in these four groups (Additional file 1: Figure S6). In the unstimulated group and the LPS group, over 90% monocytes were CD14 positive (97.0% ± 1.1% for unstimulated group and 93.1% ± 1.6% for LPS group). However, when α-LA was added, the percentage of CD14^+^ monocytes significantly decreased (49.2% ± 11.6% for LPS + α-LA group and 71.1% ± 11.3% for α-LA group), indicating the induction of anti-inflammatory nonclassical monocytes by α-LA [27].

## Discussion

The critical features of ACLF are intense systemic inflammation, pro-inflammatory precipitating events and single- or multiple-organ failures, which makes it different from acutely decompensated cirrhosis and recognized as a clinical entity [1]. The severe systemic inflammation is the main driver of widespread tissue injury and multiple-organ failures [28]. Generally, the greater the intensity of systemic inflammation, the larger the number of organ failures and the higher the short-term mortality [29]. For HBV-related ACLF specifically, the degree of systemic inflammation, which is assessed by the neutrophil–lymphocyte ratio, is also identified as an independent risk factor for ACLF development [30]. However, immune deficiency, or immune paralysis, occurs along with the intense systemic inflammation as a response to counteract inflammation [8]. As the result in this research, both the pro-inflammatory effectors (IL-1α, IL-1β, IL-6, IL-8, IFN-γ and TNF-α) and anti-inflammatory effectors (IL-4, IL-10 and IL-13) showed a trend of increase in the plasma of ACLF patients. In fact, CAID is peaked in ACLF, which manifests as a decrease or dysfunction of pro-inflammatory cells but an increase in inhibitory cells as well as anti-inflammatory effectors [31]. Monocytes from ACLF patients expressed reduced level of HLA-DR, showed impaired phagocytic capacity and low response to LPS, but secreted elevated level of IL-10 [32–34]. Meanwhile, MER receptor tyrosine kinase (MerTK)-expressing monocytes and macrophages were greatly increased in the circulation and liver of ACLF patients, and the level of MerTK expression was related to the disease severity and intensity of systemic inflammation [35]. MerTK-expressing monocytes showed reduced response to LPS, whereas MerTK inhibition restored their pro-inflammatory function, indicating MerTK-expressing monocytes as immunoregulatory cells [35]. In addition, immunosuppressive CD14^+^HLA-DR^−^ mononuclear myeloid-derived suppressor cells (M-MDSCs) expanded in ACLF patients, which had impaired monocyte function and suppress T cells [36]. As a consequence of CAID, ACLF patients were susceptible to bacterial infection, which per se was an important precipitating event for ACLF development and increased the mortality of patients [37, 38].

Although the immune deficiency of the circulating immune cells has been well-proven, the detailed immune microenvironment in the liver during ACLF has not been elucidated. As the result in this work, the liver showed less severe inflammation when compared with the periphery during ACLF. Through the technique of scRNA-seq, the immune landscape of the liver in ACLF was mapped in this research. The most significant feature in the ACLF liver was the increase and reprogramming of Mono/Mac population compared with that of cirrhosis patients and HCs. Previously, it was reported that the hepatic KCs were exhausted during acute liver injury [39, 40]. Meanwhile, the peripheral monocytes were recruited into the liver through MCP-1/CCR2 signaling and differentiated into macrophages, which leaded to the expansion of intrahepatic macrophages [41–44]. The recruited monocyte-derived macrophages (MoMFs) in the injury area played an important role in restoration and tissue-repair [40, 45], which could replace KCs when they were completely exhausted [46–49]. The results of this research confirmed the reprogramming of Mono/Mac in human livers during ACLF, in which KCs were exhausted and replaced by the recruited MoMFs. The pseudotime analysis further showed that the ACLF characterized clusters Mono1 and Mono4 were differentiated from the infiltrated monocytes but not the proliferation of KCs. The recruited MoMFs constituted the unique immune microenvironment in ACLF livers.

Cluster Mono1, which was characterized by the expression of APOC1, TREM2 and APOE, was the predominant Mono/Mac subpopulation in the ACLF liver. APOC1 was considered as an inhibitor of lipoprotein binding to the low-density lipoprotein (LDL) receptor, LDL receptor-related protein, and very low-density lipoprotein (VLDL) receptor. APOC1 was reported to interfere directly with fatty acid uptake and was also the major plasma inhibitor of cholesteryl ester transfer protein. It bound FFAs and reduced their intracellular esterification. APOC1 also modulated the interaction of APOE with beta-migrating VLDL and inhibited binding of beta-VLDL to the LDL receptor-related protein [50]. Besides, APOC1^+^ macrophages were reported as tumor-associated macrophages in hepatocellular carcinoma (HCC). Inhibition of APOC1 reversed the M2 phenotype to the M1 phenotype via the ferroptosis pathway in HCC. *APOC1*^−/−^mice showed demonstrated consistent tumor attenuation, suggesting a potential immunosuppressive phenotype of Mono1 [51].

APOE was an apolipoprotein associating with lipid particles that mainly functioned in lipoprotein-mediated lipid transport between organs via the plasma and interstitial fluids [52]. APOE was also involved in the biosynthesis by the liver of VLDLs as well as their uptake by peripheral tissues ensuring the delivery of triglycerides and energy storage in muscle, heart and adipose tissues [53]. Besides, APOE^+^TREM2^+^ macrophages were considered as tumor associated macrophages which were enriched in tumors from patients who recurred following surgery, suggesting the potential immune modulating function of APOE [54].

TREM2 was a member of triggering receptors expressed on myeloid cells (TREM) family, and belonged to the immunoglobulin superfamily receptors. Unlike TREM1 which augmented inflammation during liver injury, TREM2 acted as a natural brake on inflammation and protected liver from pathological changes [55]. Perugorria et al. reported that TREM2 was expressed on hepatic NPCs, mainly macrophages and hepatic stellate cells, during liver injury for both human and mice. The *TREM2*^*−/−*^ mice showed much more severe liver damage and inflammation in both acute liver injury and ACLF models due to augmented TLR4-driven pro-inflammatory responses [56]. Coelho et al. also confirmed the upregulation of TREM2 on macrophages in acute and chronic liver injuries. In addition, TREM2 controlled the phenotypic shifts of liver macrophages, which was from the pro-inflammatory phenotype to the restorative phenotype, and inhibited the accumulation of liver-damage associated endothelial cells [57]. In liver cirrhosis, the scar-associated macrophages, which were defined as TREM2^+^CD9^+^ macrophages, differentiated from circulating monocytes and expanded in liver fibrosis. The pro-fibrogenic effect of TREM2^+^CD9^+^ macrophages indicated that these cells contributed to restoration after liver injury [58]. In our research, TREM2 was specifically highly expressed in Mono1, thus it was selected as the marker of Mono1. The functional assessment revealed that the TREM2^+^ Mono1 were anti-inflammation phenotype macrophages and showed immunosuppressive function. The expression of TREM2 was significantly higher in M2 and correlated with the expression of IL-10 and CD206. Therefore, the TREM2^+^ Mono1 might suppress the intrahepatic inflammation and contribute to the immunosuppressive environment in the liver during ACLF.

Recent researches highlighted the fact that metabolism constructed immunity [11]. The liver was the most important organ for metabolism, and patients during ACLF suffered from metabolic disorders in addition to immune dysregulation [12]. Through metabolomics analysis, a series of metabolites in the serum or plasma were identified as biomarkers which discriminated ACLF from acute decompensation or chronic liver failure [12]. The transcriptomics analysis of PBMCs also confirmed the immune-metabolism disorder during the development of HBV-ACLF, and identified the differentially expressed genes and metabolic pathways as biomarkers for ACLF [17]. Remarkably, a critical feature of the metabolic disorders for ACLF was the disruption of lipid and fatty acid metabolism, manifesting as the upregulation of genes related to the peroxisome proliferator-activated receptor (PPAR) and mTOR signaling pathways [17]. The increase of fatty acylcarnitines and precursor of carnitine in the serum of ACLF patients also indicated the reduced mitochondrial β-oxidation, which might result in impaired energy production and organ failure [14]. In our research, we directly detected the lipid metabolites in the liver samples, and found that unsaturated FFAs associated with α-LA, α-LA metabolism and beta oxidation of very long chain fatty acids were significantly upregulated in ACLF. In vitro stimulation test with α-LA further validated the promotive effects on TREM2 expression under inflammatory environment. Our results provided direct evidence of lipid metabolic disorder in the liver of ACLF, and the abnormal accumulation of specific lipid metabolites might contribute to the unique immune microenvironment in the liver.

**Fig. 7:**
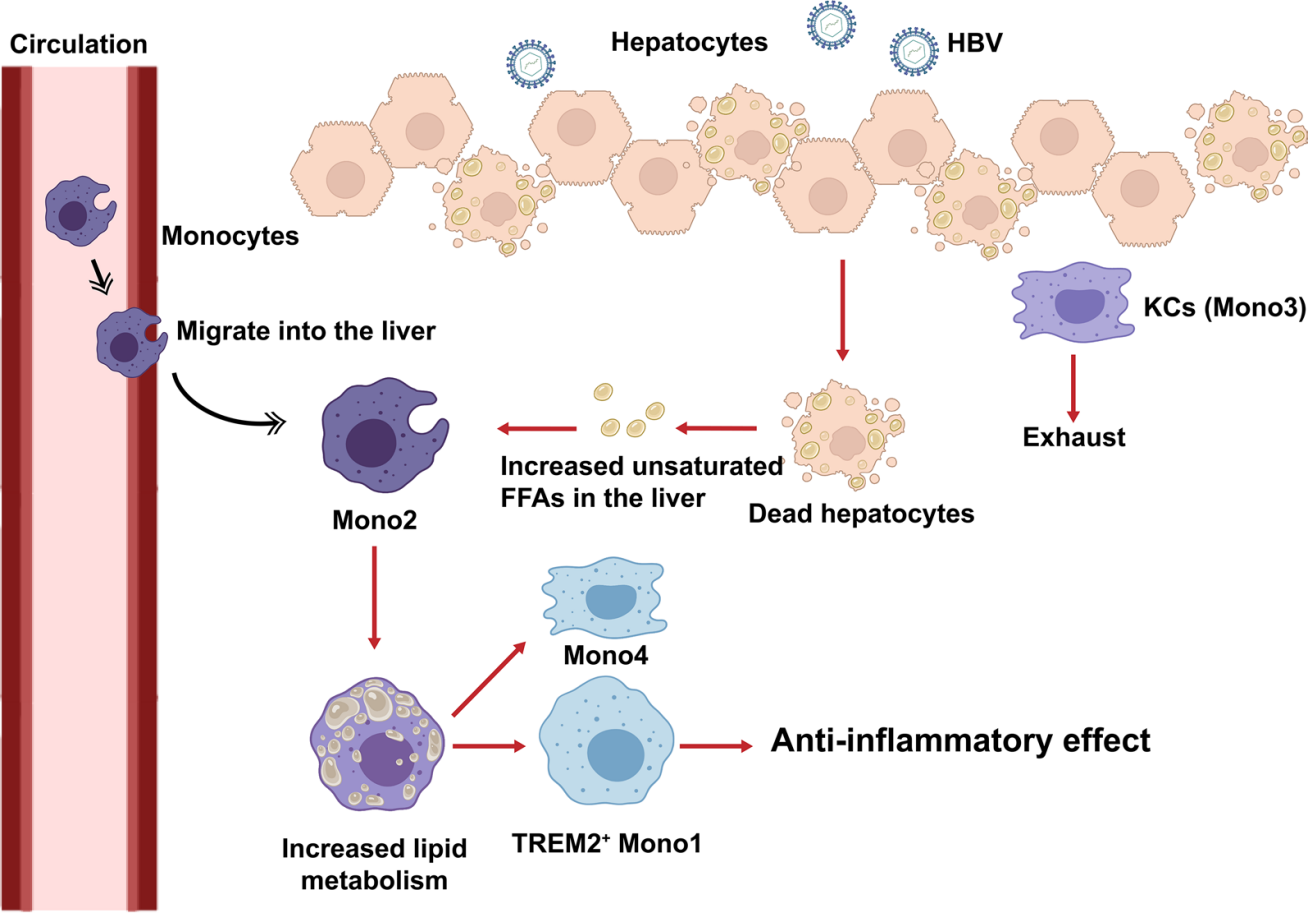
The diagram of macrophage reprogramming in the liver during ACLF. The KCs were exhausted and the peripheral monocytes were recruited into the liver during ACLF. The accumulation of unsaturated FFAs in the liver promoted macrophage reprogramming and induced the expression of TREM2 on macrophages, which contributed to the immunosuppressive microenvironment in the liver

Macrophages were a population of immune cells with high plasticity, and metabolism contributed to the reprogramming of macrophages [59, 60]. Previous studies reported that lipid metabolism could influence the differentiation and function of macrophages. Fatty acid oxidation was considered as the main metabolic way in M2 and promoted its immunoregulatory effects, whereas glycolysis was dominant in M1 [61]. In particular, the unsaturated FFAs, such as the oleic acid and α-LA, could promote transformation of M0 into M2 in vitro [62]. In contrast, the saturated FFAs activated NLRP3 inflammasome and promoted inflammation in macrophages [63]. Cluster Mono1 in our study were enriched in the expression of lipid metabolism related genes, such as *APOE, APOC1, FABP5* and *TREM2*, and these genes were upregulated along the pseudotemporal trajectory from Mono2 to Mono1, indicating the reprogramming of macrophages during ACLF was regulated by lipid metabolism. The accumulation of unsaturated FFAs in the liver during ACLF, which might be due to the massive necrosis of hepatic parenchyma cells and impairment of mitochondrial β-oxidation, promoted the differentiation of immunoregulatory macrophages infiltrated from periphery, and contributed to the immunosuppressive microenvironment in the liver (Fig. 7). Therefore, it might be a potential target to improve the immune deficiency of ACLF patients through regulating lipid metabolism.

There were some limitations of this study. The sample size of the scRNA-seq was relatively small, and only HBV-related ACLF was included for analysis. A larger, multi-center study containing different etiologies of ACLF should be performed in future to further validate the reprogramming of macrophages in ACLF livers. In addition, the effects of TREM2^+^ Mono1 on ACLF could be further explored using *TREM2* conditional knock out mouse model.

## Conclusions

In conclusion, we found the exhaustion of KCs and replacement in situ by infiltrated MoMFs in the livers of HBV-related ACLF, indicating the reprogramming of intrahepatic macrophages. The immunosuppressive TREM2^+^ macrophages were characteristically enriched in the ACLF liver and contributed to the immunosuppressive hepatic microenvironment. The accumulation of unsaturated FFAs in the ACLF liver promoted the differentiation of TREM2^+^ macrophages. Thus it might be a potential target to improve the immune deficiency of ACLF patients though regulating lipid metabolism.

## Supplementary Information

**Additional file 1: Figure S1.** The diagnostic criteria of ACLF in this study. **Figure S2.** The flow diagram for the scRNA-seq of NPCs in the livers. **Figure S3.** The basic scRNA-seq analysis showed a significant increase of Mono/Mac in ACLF livers. **Figure S4**. The KEGG and GO analyses for cluster Mono1 and Mono4. **Figure S5.** The gene enrichment analysis of the upregulated genes along the pseudotemporal trajectory from Mono2 to Mono1. **Figure S6.** The expression of CD14 on selected monocytes in stimulation test. **Table S1.** The clinical data of cirrhosis and ACLF patients. **Table S2.** The markers of proinflammatory/anti-inflammatory macrophage genes.

## Data Availability

The datasets generated and/or analysed during the current study are available in the Sequence Read Archive (SRA) repository, https://www.ncbi.nlm.nih.gov/sra/PRJNA913603.

## Abbreviations

α-LA: α-Linolenic acid
ACLF: Acute-on-chronic liver failure
CAID: Cirrhosis-associated immune dysfunction
COSSH-ACLF: Chinese Group on the Study of Severe Hepatitis B-ACLF
DAMP: Damage-associated molecular pattern
DC: Dendritic cell
DPBS: Dulbecco’s phosphate-buffered saline
EASL-CLIF: European Association for the Study of the Liver–Chronic Liver Failure Consortium
Endo/Epi: Endothelial/epithelial cells
FBS: Fetal bovine serum
FCM: Flow cytometry
FFA: Free fatty acid
GO: Gene ontology
HBV: Hepatitis B virus
HC: Healthy control
HCC: Hepatocellular carcinoma
HCV: Hepatitis C virus
IFN: Interferon
IL: Interleukin
KC: Kupffer cell
KEGG: Kyoto encyclopedia of genes and genomes
LDL: Low-density lipoprotein
LPS: Lipopolysaccharide
MACS: Magnetic activated cell sorting
MCP-1: Monocyte chemoattractant protein-1
MerTK: MER receptor tyrosine kinase
MFI: Median fluorescence intensity
M-MDSC: Mononuclear myeloid-derived suppressor cell
MoMF: Monocyte-derived macrophage
Mono/Mac: Monocytes/macrophages
MRC-1: Mannose receptor C-1
NAFLD: Non-alcoholic fatty liver disease
Neu: Neutrophil
NPC: Non-parenchymal cell
PAMP: Pathogen-associated molecular pattern
PBMC: Peripheral blood mononuclear cell
PBS: Phosphate-buffered saline
PMA: Phorbol 12-myristate 13-acetate
PPAR: Peroxisome proliferator-activated receptor
scRNA-seq: Single-cell RNA-sequencing
SD: Standard deviation
SEM: Standard error of mean
TNF: Tumor necrosis factor
TREM: Triggering receptors expressed on myeloid cells
UMI: Unique molecular identifier
UPLC-MS/MS: Ultra-performance liquid chromatography-tandem mass spectrometry
VLDL: Very low-density lipoprotein
