# Supplementary material for: Intrahepatic macrophage reprogramming associated with lipid metabolism in hepatitis B virus-related acute-on-chronic liver failure
Source: J Transl Med. 2023 Jun 28;21:419. doi: 10.1186/s12967-023-04294-1 (PMC10303321; doi:10.1186/s12967-023-04294-1)
Supplement: Supplementary file 1 — Additional file 1: Figure S1. The diagnostic criteria of ACLF in this study. Figure S2. The flow diagram for the scRNA-seq of NPCs in the livers. Figure S3. The basic scRNA-seq analysis showed a significant increase of Mono/Mac in ACLF livers. Figure S4. The KEGG and GO analyses for cluster Mono1 and Mono4. Figure S5. The gene enrichment analysis of the upregulated genes along the pseudotemporal trajectory from Mono2 to Mono1. Figure S6. The expression of CD14 on selected monocytes in stimulation test. Table S1. The clinical data of cirrhosis and ACLF patients. Table S2. The markers of proinflammatory/anti-inflammatory macrophage genes. [file 12967_2023_4294_MOESM1_ESM.docx]

**Additional file**

1. **
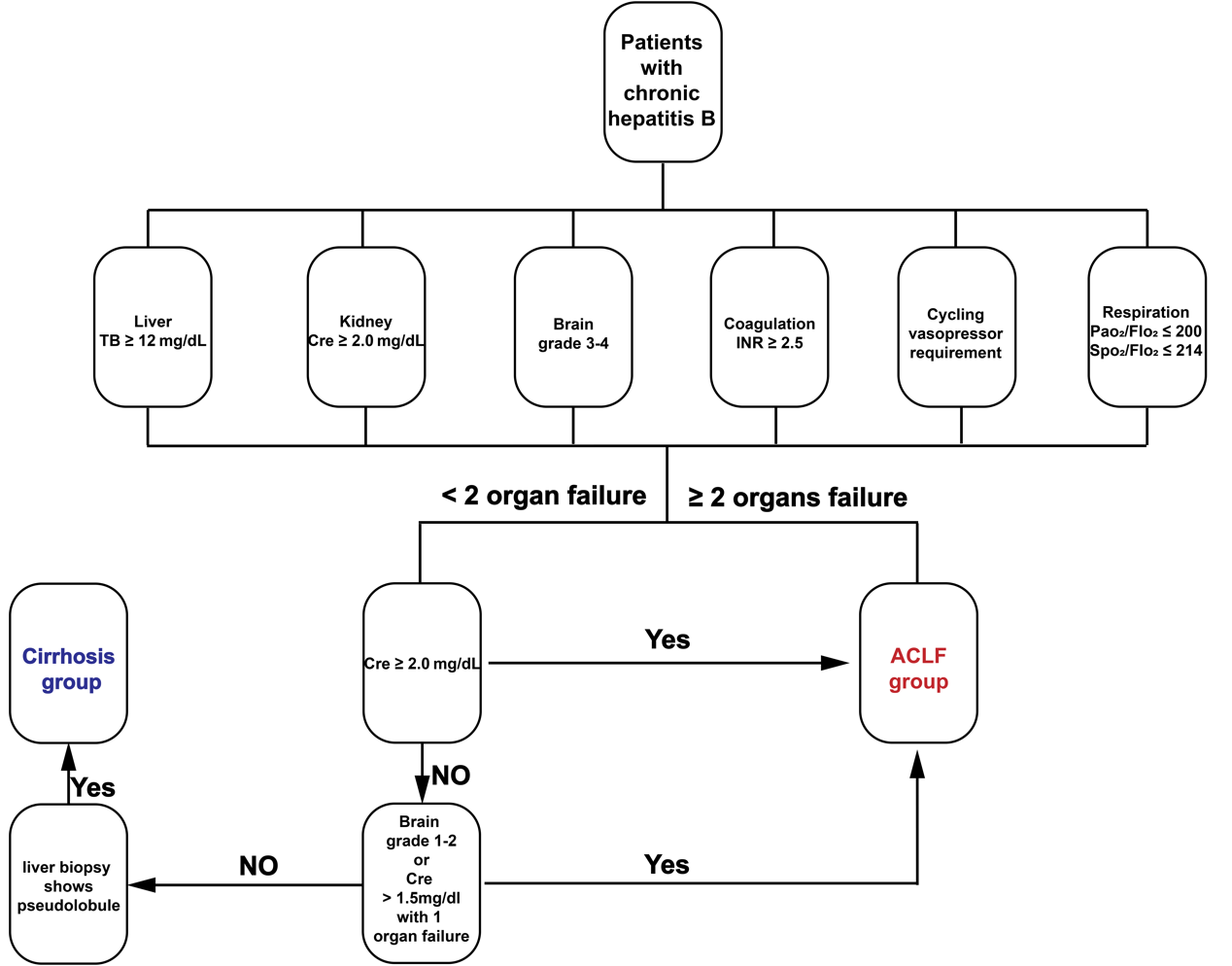
Additional file figures.**

**Additional file figure 1** The diagnostic criteria of ACLF in this study according to the definition of European Association for the Study of the Liver-Chronic Liver Failure Consortium (EASL-CLIF). The recruited patients also met the definition of the Chinese Group on the Study of Severe Hepatitis B-ACLF (COSSH-ACLF), which was defined as single liver failure (TB ≥ 12mg/dL) and an INR ≥ 1.5 regardless of the presence of cirrhosis.


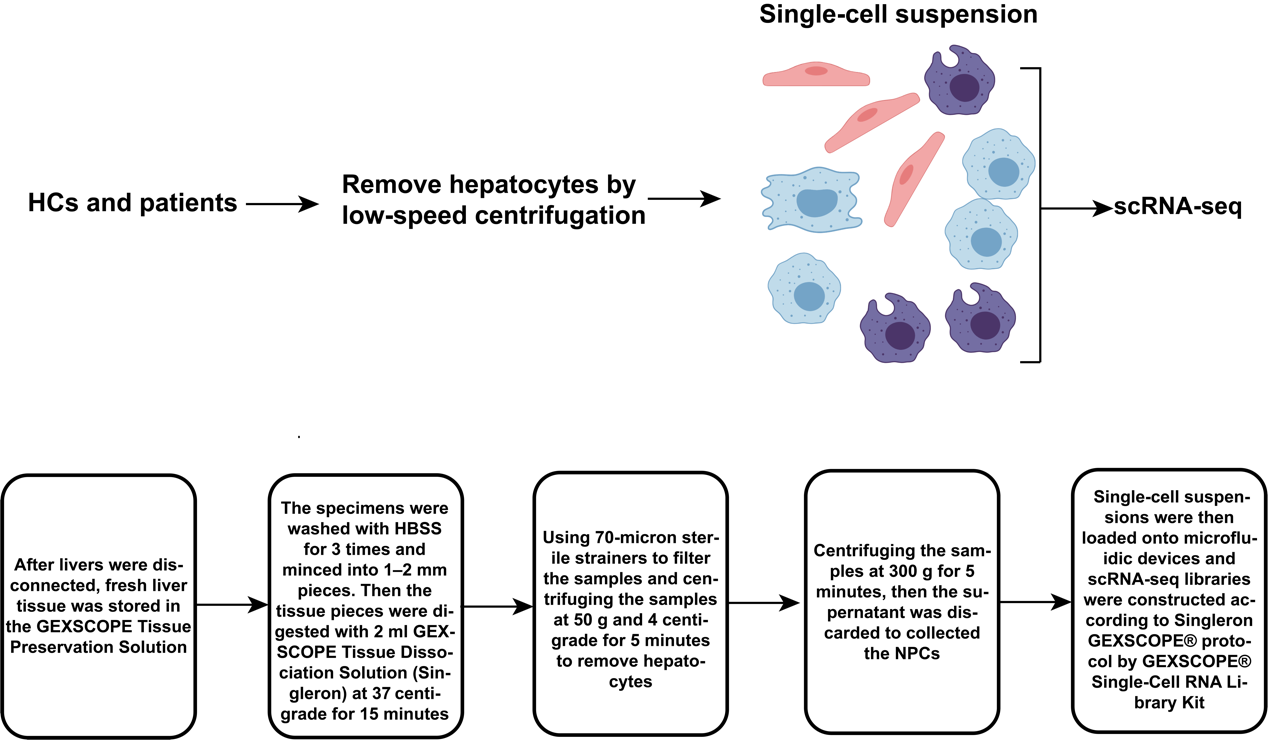


**Additional file figure 2** The flow diagram for the scRNA-seq of NPCs in the livers.


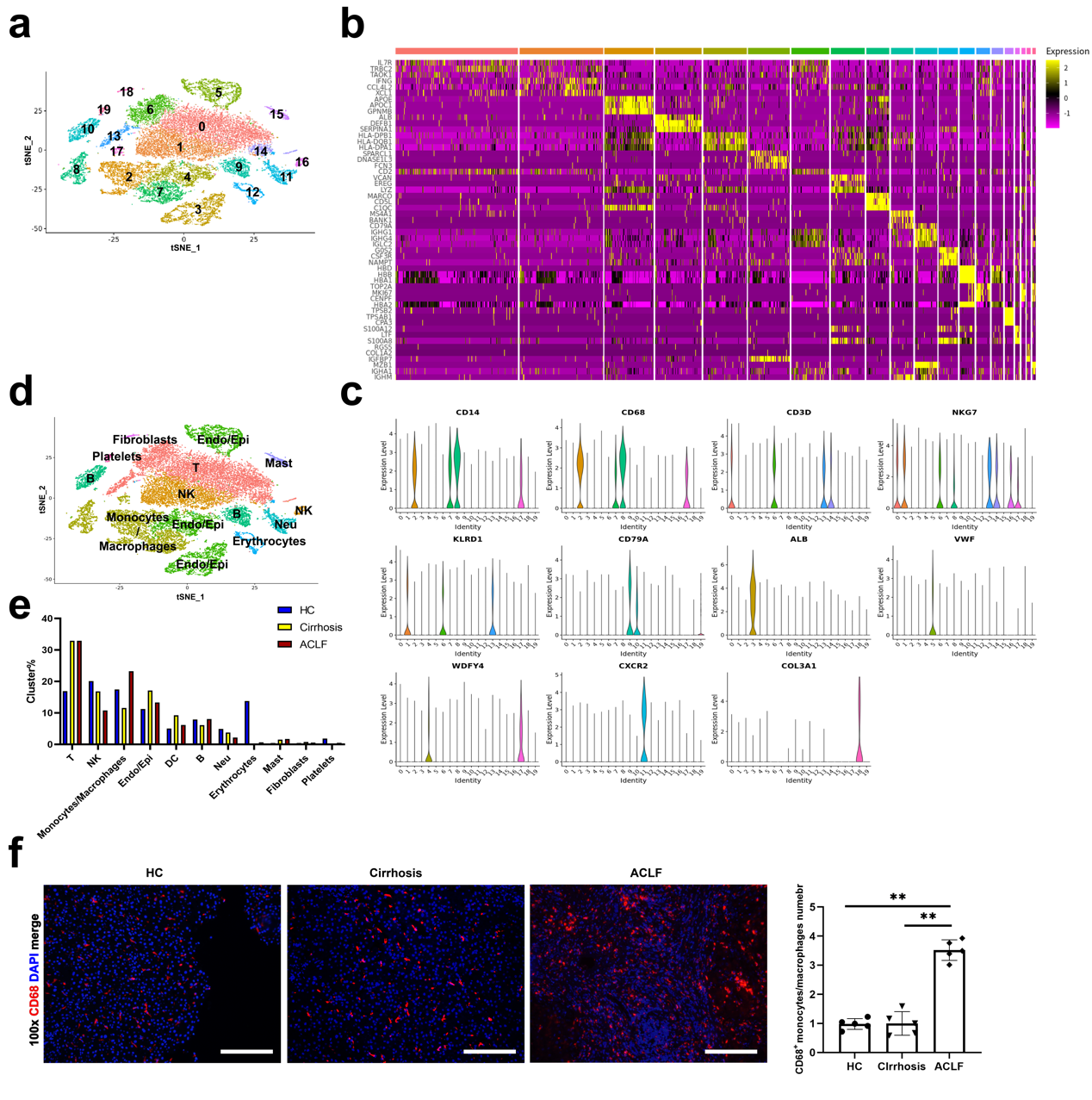


**Additional file figure 3 a** 26,200 cells from HCs (n = 2), cirrhosis (n = 3) and ACLF (n = 5) human livers were clustered into 20 subpopulations (0 – 19). **b** The heat map of representative marker genes for the 20 subpopulations which were arranged in the order of 0 – 19. **c** The classical marker genes of each cluster. **d** The 20 subpopulations were re-annotated into 11 classical clusters according to their marker genes. **e** The proportions of 11 classical clusters among HCs, cirrhosis and ACLF patients. **f** Immunofluorescence staining for monocytes/macrophages (CD68) in the livers of HCs, cirrhosis and ACLF patients (100× field, ** p ＜ 0.01, bar = 200 μm). The scRNA-seq data and figures a – e were cited from our previous work (Ref 18, DOI: 10.3390/jcm11102910).

**
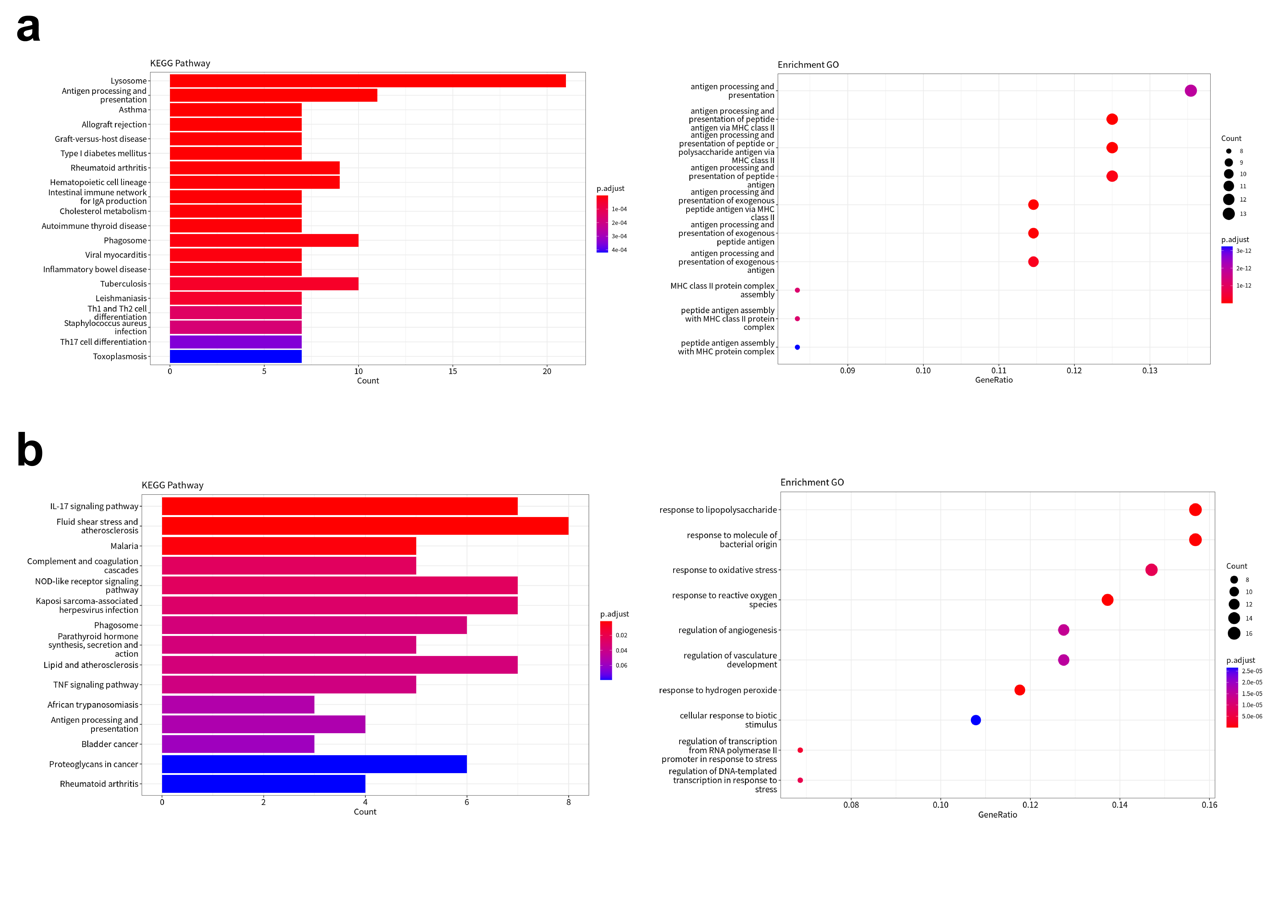
Additional file figure 4 a** The KEGG and GO analyses for cluster Mono1. **b** The KEGG and GO analyses for cluster Mono4.


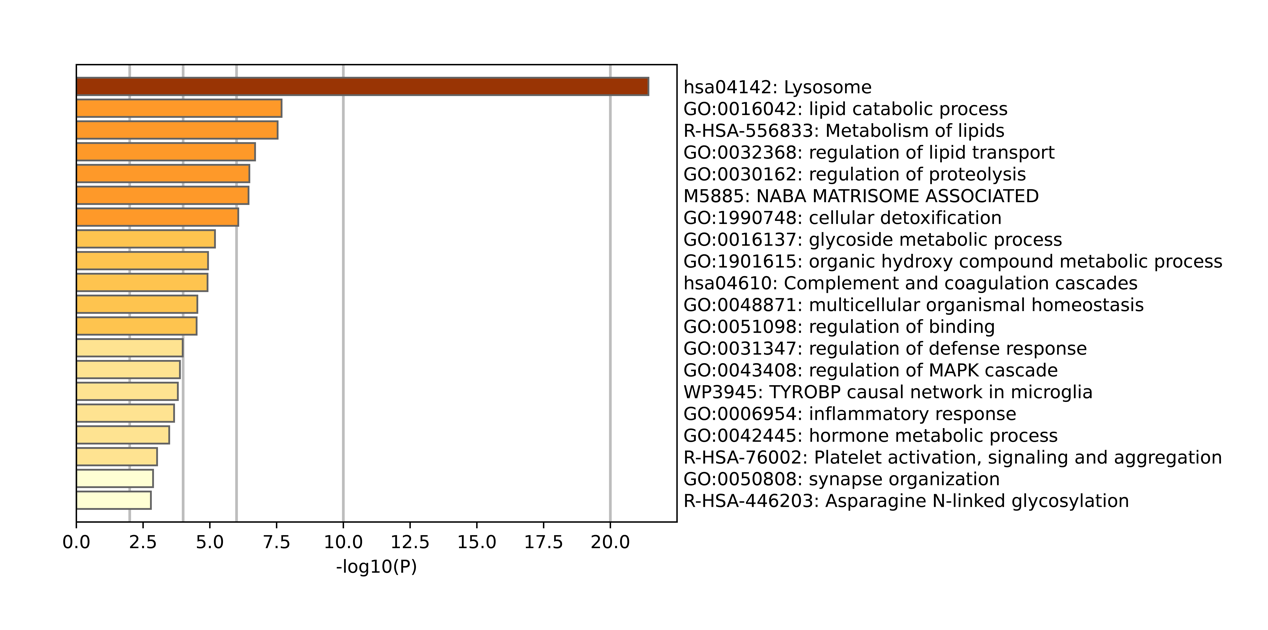


**Additional file figure 5** The gene enrichment analysis of the upregulated genes along the pseudotemporal trajectory from Mono2 to Mono1.


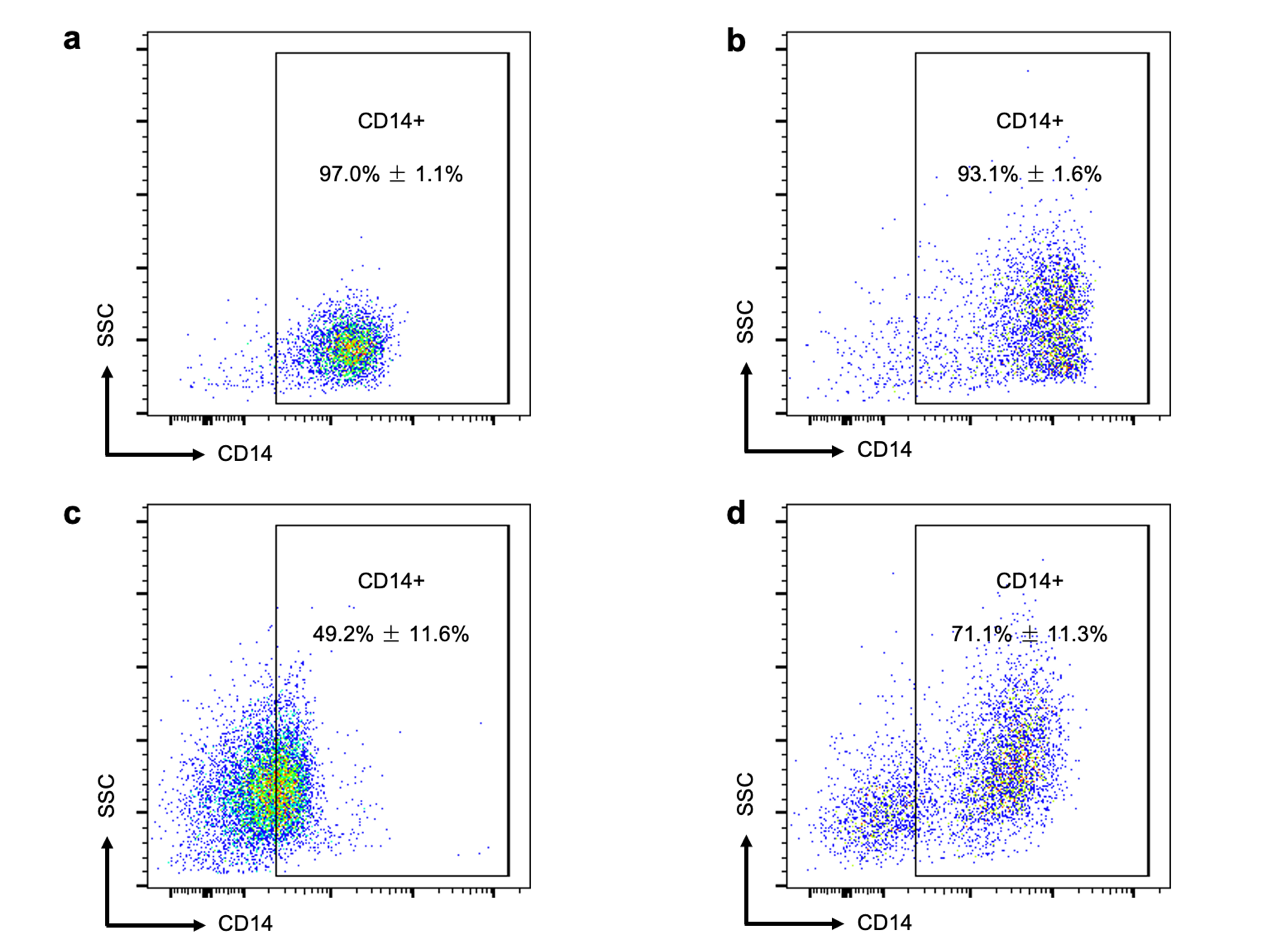


**Additional file figure 6** The expression of CD14 on selected monocytes in stimulation test. **a** unstimulated group; **b** LPS group; **c** LPS + α-LA group; **d** α-LA group.

1. **Additional file tables.**

| **Additional file Table 1. The clinical data of cirrhosis and ACLF patients** | | | | | | | | | | | | | | | | | | |
| --- | --- | --- | --- | --- | --- | --- | --- | --- | --- | --- | --- | --- | --- | --- | --- | --- | --- | --- |
| **Number** | **Gender** | **Age (year)** | **Group** | **Na (mmol/L)** | **Liver** | | | | **Kidney** | | **Brain** | **Coagulation** | | **Circulation** | **Respiration** | **ACLF score^*^** | **ACLF grade^*^** | **MELD-Na score** |
|  |  |  |  |  | ALT (U/L) | AST (U/L) | Tbil (umol/L) | Score | Creatinine (umol/L) | Score | Score | INR | Score | Score | Score |  |  |  |
| 1 | Male | 46 | Cirrhosis | 144 | 28 | 32 | 52.5 | 1 | 74 | 1 | 1 | 1.4 | 1 | 1 | 1 | 6 | Absence | 14 |
| 2 | Male | 37 | Cirrhosis | 132 | 59 | 59 | 41.7 | 1 | 39 | 1 | 1 | 1.4 | 1 | 1 | 1 | 6 | Absence | 18 |
| 3 | Male | 54 | Cirrhosis | 143 | 73 | 63 | 27.4 | 1 | 165 | 1 | 1 | 1.1 | 1 | 1 | 1 | 6 | Absence | 15 |
| 4 | Male | 45 | ACLF | 148 | 457 | 728 | 446.1 | 3 | 90 | 1 | 1 | 2.7 | 3 | 1 | 2 | 11 | ACLF-2 | 30 |
| 5 | Male | 37 | ACLF | 138 | 67 | 82 | 576.7 | 3 | 78 | 1 | 3 | 3.1 | 3 | 1 | 1 | 12 | ACLF-3 | 31 |
| 6 | Male | 52 | ACLF | 133 | 141 | 91 | 387.2 | 3 | 52 | 1 | 1 | 3.6 | 3 | 1 | 2 | 11 | ACLF-2 | 33 |
| 7 | Male | 48 | ACLF | 144 | 384 | 221 | 306.4 | 3 | 55 | 1 | 1 | 3.0 | 3 | 1 | 2 | 11 | ACLF-2 | 30 |
| 8 | Male | 57 | ACLF | 139 | 1704 | 2677 | 339.9 | 3 | 76 | 1 | 1 | 3.0 | 3 | 1 | 1 | 10 | ACLF-2 | 30 |
| ^*^ The score and grade of ACLF are assessed according to the EASL-CLIF Consortium organ-failure scoring system. ACLF, acute-on-chronic liver failure. ALT, alanine aminotransferase. AST, aspartate aminotransferase. Tbil, total bilirubin. INR, international normalized ratio. MELD, model for end-stage liver disease. EASL-CLIF, European Association for the Study of the Liver–Chronic Liver Failure. | | | | | | | | | | | | | | | | | | |

**Additional file table 2. The markers of proinflammatory/anti-inflammatory macrophage genes**

| **Pro-inflammation** | **Anti-inflammation** |
| --- | --- |
| CD40 | TREM2 |
| PTGS2 | CHIT1 |
| IL12A | CHI3L1 |
| IL12B | APOC1 |
| ICAM1 | LIPA |
| IFIT2 | APOC2 |
| VCAM1 | APOE |
| RSAD2 | GM2A |
| GPR84 | CTSD |
| NFKBIZ | IFI27 |
| EHD1 | ACP5 |
| TRAF1 | FABP5 |
| CCND2 | GPNMB |
| SOCS3 | RNASE1 |
| ADORA2A | GCHFR |
| SERPINE1 | CD9 |
| SERPINB2 | LPL |
| CD38 | CTSZ |
| INHBA | C1QB |
| LCN2 | VAT1 |
| MEFV | PLD3 |
| NFKB2 | CYP27A1 |
| IL15RA | MARCO |
| IRF7 | IFI6 |
| ZBP1 | FTL |
| PILRA | MSR1 |
| OAS3 | LGALS3 |
| IFIH1 | A2M |
| SLCO3A1 | NUPR1 |
| NFKBIA | CAPG |
| MET | CD81 |
| ISG20 | CD59 |
| STAT2 | CTSB |
| BATF2 | LGMN |
| TLR2 | GRN |
| CFLAR | SPARC |
| HDC | PLA2G7 |
| NUPR1 | HEXB |
| GAS7 | DBI |
| MMP14 | ACE |
| DHX58 | PRDX1 |
| AGRN | CTSL |
| VCAN | GSN |
| IFI35 | ANXA2 |
| JAK2 | LGALS3BP |
| ARG2 | PSAP |
| GCH1 | SCD |
| PELI1 | C1QA |
| DDX60 | LY6E |
| VASP | CSTB |
| ZUFSP | LAMP1 |
| EIF2AK2 | C1QC |
| SOD2 | BCAP31 |
| CD274 | GALM |
| ELL2 | SERPING1 |
| PTGES | CTSK |
| BATF | CD63 |
| SLC25A37 | TIMP2 |
| PSTPIP2 | ABHD12 |
| ITGAL | TNS3 |
| PARP9 | RARRES1 |
| NOTCH1 | RMDN3 |
| HCK | SRGN |
| SNX20 |  |
| NFKBIB |  |
| CCRL2 |  |
| SAMSN1 |  |
| DUSP2 |  |
| CD14 |  |
| CXCL16 |  |
| JDP2 |  |
| TAPBPL |  |
| STAT1 |  |
| RBPMS |  |
| RAB32 |  |
| MAP3K5 |  |
| IL1RN |  |
| FLNB |  |
| BIRC3 |  |
| TAP2 |  |
| MITD1 |  |
| GGCT |  |
| TNIP1 |  |
| CD86 |  |
| IRF9 |  |
| IL17RA |  |
| TRIM25 |  |
| LMO4 |  |
| ACSL1 |  |
| EBI3 |  |
| GRAMD1A |  |
| ITGA5 |  |
| RNF114 |  |
| AGTRAP |  |
| PSMB9 |  |
| IFNAR2 |  |
| ST3GAL3 |  |
| SLC31A1 |  |
| TNFRSF1B |  |
| MTDH |  |
| CPD |  |
| PSMB8 |  |
| SKIL |  |
| PDPN |  |
| SNX10 |  |
| SYK |  |
| DENR |  |
| GADD45B |  |
| SLC15A3 |  |
